# Microsurgical Clipping of Carotid-Ophthalmic Tandem Aneurysms: Case Report and Surgical Nuances

**DOI:** 10.3390/medicina57070731

**Published:** 2021-07-19

**Authors:** Matias Costa, Matías Baldoncini, Zachary L. Tataryn, Mickaela Echavarria Demichelis, Agustin Conde, Cynthia Purves, Alice Giotta Lucifero, Juha Hernesniemi, Sabino Luzzi

**Affiliations:** 1 Cerebrovascular Neurosurgery Department, Swedish Neuroscience Institute, Seattle, WA 98122, USA; matiascostamd@gmail.com; 2 Department of Neurological Surgery, Hospital San Fernando, Buenos Aires 1646, Argentina; drbaldoncinimatias@gmail.com (M.B.); mickaelaechavarria@hotmail.com (M.E.D.); ag_conde@hotmail.com (A.C.); 3 Spine Department, Swedish Neuroscience Institute, Seattle, WA 98122, USA; zactataryn@gmail.com; 4 Division of Interventional Neuroradiology, Juan A. Fernandez Hospital and Güemes Clinic, Buenos Aires C1425 CABA, Argentina; cynthiapurves@gmail.com; 5 Neurosurgery Unit, Department of Clinical-Surgical, Diagnostic and Pediatric Sciences, University of Pavia, 27100 Pavia, Italy; alicelucifero@gmail.com; 6 Juha Hernesniemi International Center for Neurosurgery, Henan Provincial People’s Hospital, Zhengzhou 450003, China; juha.hernesniemi@icloud.com; 7 Neurosurgery Unit, Department of Surgical Sciences, Fondazione IRCCS Policlinico San Matteo, 27100 Pavia, Italy

**Keywords:** anterior clinoidectomy, carotid-ophthalmic aneurysms, clipping, flow-diverter, internal carotid artery, tandem intracranial aneurysms

## Abstract

Tandem intracranial aneurysms (TandIAs) are rare but inherently complex, and special technical considerations are required for their surgical management. The present case highlights the key surgical aspects of two carotid-ophthalmic TandIAs incidentally found in a 60-year-old female. Both the aneurysms were superiorly projecting, regular in size, and involved the left ophthalmic segment of the internal carotid artery (ICA). The minimum distance between the necks was 3 mm. The patient underwent microsurgery because of the reported major complications rate of the endovascular treatment in the case of a very short minimum distance between the TandIAs. After cervical ICA exposure, both the aneurysms were excluded through a pterional approach. Intradural anterior clinoidectomy and unroofing of the optic canal allowed the mobilization of the left optic nerve. The more distal aneurysm was clipped before the opening of the distal dural ring of the ICA. The proximal aneurysm was clipped with two straight clips stacked perpendicular to the ICA. A small remnant was intentionally left to avoid the stenosis of the ophthalmic artery. Postoperative angiography showed the exclusion of both the aneurysms with a small dog-ear of the more proximal one. The patient was discharged neurologically intact and, after one year, the remnant remained stable. Microsurgical clipping is a definitive and durable treatment for carotid-ophthalmic TandIAs. In the case of a very short minimum distance between the aneurysms, the distal one should be clipped first to make the anterior clinoidectomy, opening of the distal dural ring of the ICA, and clipping of the more proximal aneurysm easier.

## 1. Introduction

Multiple intracranial aneurysms have been reported to have a prevalence ranging between 15–35% in the largest series [1,2,3,4,5]. Their incidence is similar across the countries worldwide but significantly higher in women, patients with autosomal dominant polycystic kidney disease, a family history of intracranial aneurysm and subarachnoid hemorrhage, brain tumor, pituitary adenoma, and atherosclerosis [5,6,7]. Tandem intracranial aneurysms (TandIAs) represent a specific angioarchitectural type, in which two aneurysms of at least 30mm in maximal diameter are nearby on the same parent vessel or two separate but not interconnected adjacent arteries. Their pathogenesis remains unclear to date, although the segmental duplication of the parent artery, also known as “fenestration” and resulting from an embryonic error, has been linked with the incidence of TandIAs [8,9,10,11,12,13,14]. TandIAs involve the internal carotid artery (ICA) in 87% of cases, and specifically the ophthalmic (C6) segment according to Bouthillier classification in 50% of occurrences [15,16]. Other possible sites in decreasing order of frequency are the vertebral artery, middle cerebral artery, basilar artery, posterior inferior cerebellar artery, anterior cerebral artery, and anterior communicating artery [15,17,18,19,20,21,22]. TandIAs may also be “kissing” in the case of a partial adhesion between aneurysmal walls [23,24]. The frequency of TandIAs has been estimated to be up to 2.8% of all intracranial aneurysms [25,26], and specific risk factors for their development, such as fenestrated arterial variants [27] and family history [28], have been suggested. In comparison with solitary aneurysms, TandIAs pose special challenges from the surgical and endovascular standpoints for several reasons: unpredictable hemodynamic effects which treating one aneurysm may have on the other one, technical difficulties associated with a possible “kissing” configuration, the need to balance the choice between single versus multiple treatment sessions, increased risk of complications resulting from the cumulative sum of morbidity rates associated with each aneurysm, and a relative lack of overall experience due to the very small number of cases reported in the literature. Accordingly, the best treatment strategy of TandIAs remains controversial despite the tremendous and constant improvement and refinement of endovascular and surgical techniques.

The purpose of this case report is to describe the technical nuances of the microsurgical management of two carotid-ophthalmic tandem aneurysms.

## 2. Case Presentation

**Figure 1:**
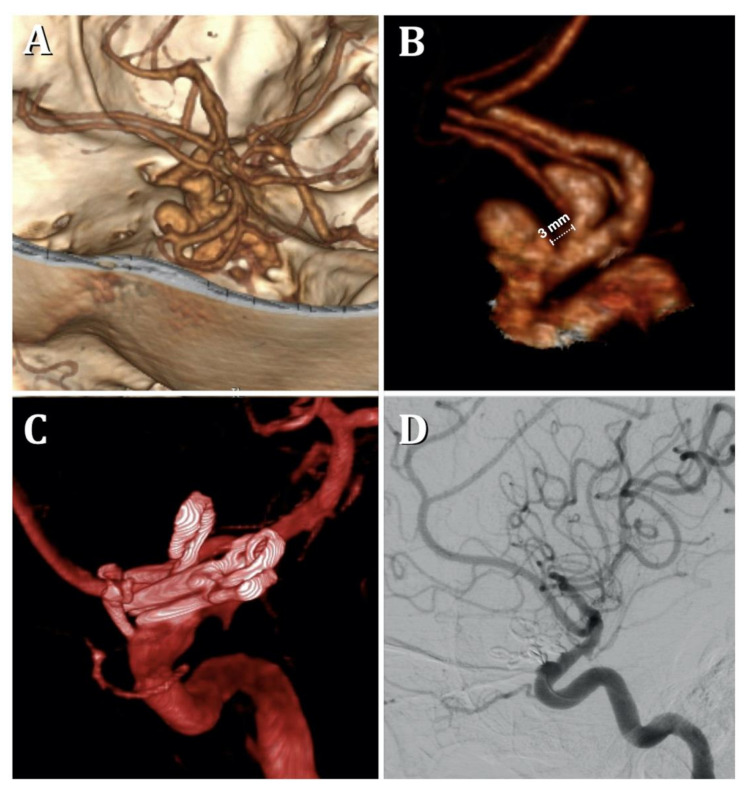
(**A**,**B**) Preoperative 3D CT angiography showing the left tandem paraclinoid internal carotid artery aneurysms. Postoperative 3D (**C**) and 2D (**D**) digital subtraction angiography in lateral projection.

A 60-year-old female with a history of systemic arterial hypertension was diagnosed with two incidental side-wall paraclinoid TandIAs of the left ICA after having undergone a 3D CT angiography due to an isolated transitory ischemic attack. Both the aneurysms were saccular, superiorly projecting, and involved the ophthalmic (C6) segment of the ICA. The size, aspect ratio, and flow angle were 8 mm, 1.5, and 92°, respectively, in the proximal aneurysm, and 7 mm, 1.7, and 110° correspondingly in the distal one (Figure 1A,B). The minimum distance between the aneurysmal necks, namely the distance between the distal end of the proximal aneurysm and the proximal end of the distal one, measured 3 mm. An MRI excluded intraluminal thromboses. Given the relatively small size of both aneurysms, the risk of intraoperative ICA sacrifice was considered negligible, and a balloon test occlusion was deemed unnecessary. Campimetry revealed a normal visual acuity, and oculomotion was intact. After a multidisciplinary neurovascular meeting, open surgical clipping of both aneurysms was decided.

**Figure 2:**
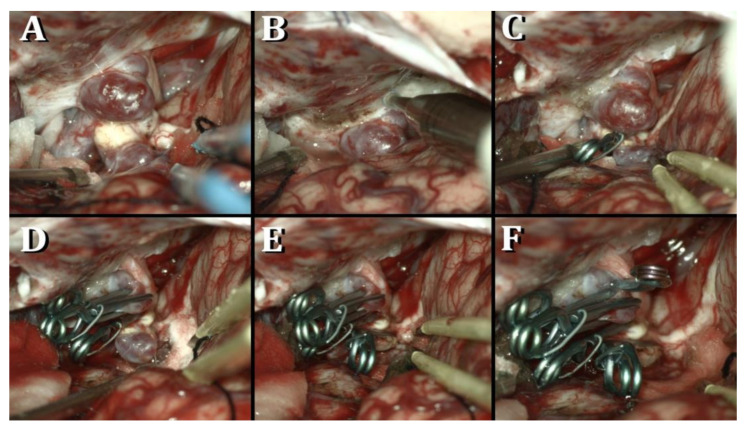
(**A**) Aneurysm’s exposure; (**B**) anterior clinoidectomy; (**C**–**F**) clipping of the tandem paraclinoid internal carotid artery aneurysms.

The exposure of the cervical ICA was accomplished to achieve proximal control before performing a left standard pterional approach with the patient’s head rotated 45° to the right side and tilted 10° in extension. Distal-to-proximal Sylvian fissure dissection was performed starting at the anterior Sylvian point. The Sylvian, carotid, optic, and chiasmatic cisterns were widely opened, and cerebrospinal fluid was released for brain relaxation. Both aneurysms were exposed in the left paraclinoid region. They presented multiple blebs, and the distal one showed a calcified neck. The left optic nerve was displaced by the proximal aneurysm. A left intradural anterior clinoidectomy and unroofing of the optic canal were performed with a 3mm diamond drill. The falciform ligament was sharply divided to gently mobilize the optic nerve. A pilot clip was initially used for the distal aneurysm. Afterward, the opening of the distal dural ring was completed before the mobilization of the left ICA. This maneuver allowed for the visualization of the take-off of the left ophthalmic artery (OphA). Multiple clipping attempts of the proximal aneurysm were executed, each of which caused stenosis at the origin of the OphA. As a consequence, the proximal aneurysm was partially obliterated with two stacked 9mm straight clips, and a small remnant was intentionally left. The distal aneurysm was definitively excluded with a 7mm angulated clip before the removal of the pilot clip (Figure 2). Post-operative angiography confirmed the complete exclusion of the distal aneurysm and a small remnant of the proximal one (Figure 1C,D). The patient was discharged without deficit on the third post-operative day, and, on angiography performed at the first-year follow-up, the known remnant of the proximal aneurysm remained stable.

## 3. Discussion

The technical considerations of the microsurgical management of two ophthalmic tandem aneurysms of the ICA have been herein reported through the description of an illustrative case suggesting several points of discussion, the first of which is the reasons for the indication for surgery.

Literature about the management of TandIAs is largely based on very small series or even case reports, which implies a lack of universal recommendations about the management of these particular types of aneurysms. In the majority of cases, the indication for surgical or endovascular treatment is primarily based on the experience of the treating team.

In the present case, microneurosurgery was considered the best treatment option because of several factors set out below. The largest series (145 patients) about the endovascular treatment of TandIAs reported by Feng and colleagues proved that a shorter minimum distance between the aneurysms, multiple-session treatment, and diabetes were independent predictors of treatment-related complications, a lower occlusion rate, and a poor outcome on multivariate regression analysis [15]. Especially regarding the short minimum distance between the aneurysms, similar data were reported by Mut and colleagues based on a computational model [29]. It should be highlighted that in the reported case, the short minimum distance between the neck of the aneurysms was just 3 mm. A further point in favor of microsurgery was the involvement of the OphA, whose occlusion risk by the side of the flow diverting stents rests unclear [30,31,32,33,34,35,36,37,38]. Adeeb et al. also stressed as larger aneurysms size negatively affects the occlusion rate of TandIAs treated with a single pipeline embolization device [19].

Despite the patient had suffered from a transitory ischemic attack, microneurosurgery was considered not at a greater risk of complications compared with endovascular treatment. In fact, in a multicenter clinical study, Qureshi and colleagues demonstrated an equal risk of major stroke between the surgical and conservative treatment [39]. Similar data were reported by McLaughlin et al. [40].

This treatment paradigm also comes from the authors’ experience in the elective surgical management of isolated paraclinoid aneurysms. Current indications for microneurosurgery of paraclinoid aneurysms in the endovascular era include broad-based large and giant ophthalmic aneurysms in young patients, prohibitive tortuosity of the cervical ICA, resistance to aspirin, or clopidogrel/prasugrel, warning syndromes, and patient preference. As a consequence of their proximity, a single-stage pipeline procedure has been suggested as the best choice for two or even more TandIAs arising from the same parent vessel [17,18,19,20,21,41,42]. Nevertheless, the reported overall aneurysmal occlusion following pipeline and coil embolization is 58.8% and 77.4%, respectively [15], and these rates are largely inferior to those of the treated solitary aneurysms with similar sizes and locations. Not surprisingly, multiple endovascular treatment sessions are associated with a worse outcome as a consequence of the cumulative morbidity risk [15]. These factors put into question the theoretical advantages of the endovascular treatment of tandem paraclinoid aneurysms. A further issue regards the full versus partial treatment in those cases where one of the two (or more) TandIAs finds no indication for treatment because of small size (<5mm). The overall risk of complications seems to not be increased for endovascular therapy if the minimum distance between the aneurysms is greater than 5 mm [15]. Conversely, no conclusive data are reported in the literature in regards to the microsurgical clipping of TandIAs.

**Figure 3:**
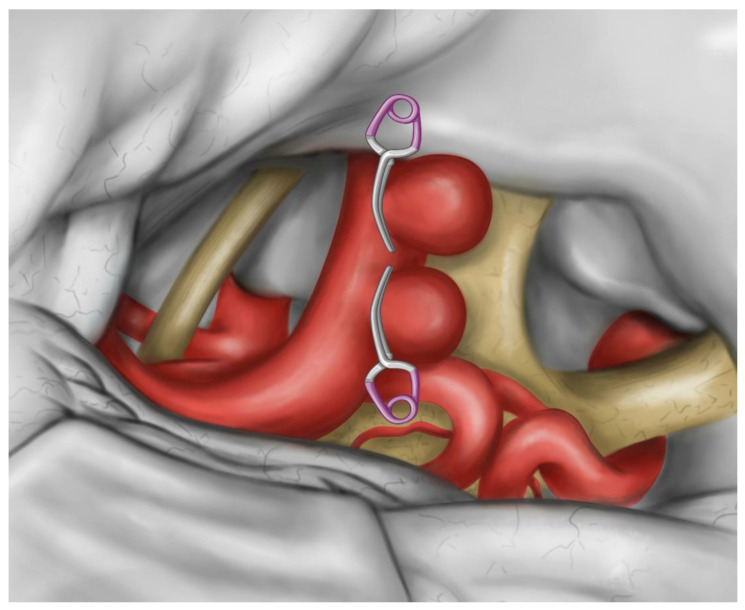
A sketch of counterclipping in the “facing” fashion.

Based on the reported pieces of evidence, the surgical risk of solitary paraclinoid aneurysms is higher in the case of giant sizes, thrombosis, or heavily calcified neck. In these cases, bypass ought to be considered [43,44,45]. A further adjunctive risk may come from the necessity to treat a blister-like aneurysm of the ICA given their aggressive natural history, morbidity, and mortality [46,47,48,49,50,51,52]. In the case of large or giant aneurysms, a balloon test occlusion of the parent vessel and is mandatory, and angiography may provide precious details to consider during treatment planning. The proximal control of the ICA for temporary occlusion is mandatory for tandem paraclinoid aneurysms, likewise to what is required in paraclinoid aneurysms at large. Different techniques are today available for this purpose. They comprehend cervical or petrous ICA exposure, extradural or intradural clinoid ICA exposure, endovascular control with balloon-assisted occlusion or suction decompression (Dallas technique), adenosine-induced cardiac standstill, and intracavernous control [53,54,55,56,57,58,59,60,61,62]. Each of these techniques has advantages and disadvantages. The exposure of the petrous ICA may be challenging or ineffective [61,63,64,65,66]. Endovascular control im-poses an additional endovascular team or hybrid operative room and is not free from a non-negligible risk of embolic strokes and intimal dissection. Cardiac standstill has been reported to be burdened by a high mortality and morbidity rate. Intracavernous control, according to Krisht’s technique, is an elegant and valuable option. Nevertheless, it needs extensive experience along with perfect knowledge of the microsurgical anatomy of the cavernous sinus. Because of their reliability, the techniques of exposure of the cervical and clinoid segments of the ICA are still today the most utilized by a large part of surgeons, including the authors. While admitting the high reliability of the extradural and intradural exposure of the clinoid segment of the ICA for the majority of small and regular paraclinoid aneurysms, we often prefer the cervical ICA in large, giant, and complex ones because of a theoretically higher risk of rupture during the anterior clinoidectomy. Considering their inherent complexity, tandem paraclinoid aneurysms as that herein reported fall into this group. A further aspect regards the reported higher risk of visual morbidity for carotid cave aneurysms and the temporary occlusion of the ICA executed at the level of the carotid cave [67]. From a technical standpoint, the surgical management of paraclinoid TandIAs in a single stage involves some additional difficulties in comparison with solitary aneurysms. A critical surgical decision arises in planning to treat the proximal or distal aneurysm first. We believe distal aneurysms ought to be excluded before attempts at proximal aneurysm occlusion for two reasons: First, a lower inflow rate, flow velocity, and wall shear stress in the more distal aneurysm affects the convex side of the ICA [29], making it less prone to intraoperative rupture. Second, the clipping of the clinoidal or ophthalmic aneurysms imposes the need for an anterior clinoidectomy, the opening of the distal dural ring, and mobilization of the ICA to allow for an effective clip placement. This avoids the sling effect and minimizes the risk of ICA stenosis after clipping [44]. All these maneuvers are hampered and complicated by the presence of a further distal aneurysm of the ophthalmic (C6) or communicating (C7) segment of the ICA [16]. Large or giant aneurysms of the dorsal ICA wall could also hinder the so-called “clinoidal cone” [68]. It must be stressed that “kissing” TandIAs represent an exception since the dissection at the ‘‘kissing point’’ and clipping of the distal aneurysm involves a tremendous risk of rupture of the proximal one, as reported by Yasargil [46]. A short distance between the necks of tandem aneurysms represents a challenging factor also for both microsurgical clipping and endovascular therapy. For both superior and inferior projecting aneurysms, tandem clipping and counter clipping in a cross-wise fashion is often technically impossible because the length of the clip line created by the blades of the overlapping clip is generally insufficient to exclude both the necks. Counterclipping in a “facing” fashion is certainly more suitable (Figure 3).

A good option to avoid the footprint of the clips is the use of stacked clips having an orientation perpendicular to the long axis of the ICA, as performed in our reported case. The parallel stacked clips are particularly useful in case of the shorter distance between the aneurysms. In the case of TandIAs having different projections, more complex clipping techniques are necessary. Our and other groups have previously stressed the role of indocyanine green and fluorescein video angiography, micro-Doppler ultrasonography, Doppler flowmetry, endoscopic assistance, and intraoperative digital subtraction angiography in neurovascular surgery [69,70,71,72,73,74,75,76,77,78,79], and we believe these tools prove critical in the management of TandIAs.

It must be stressed that larger series of TandIAs are necessary to validate the technical tips used in the management of the present case.

## 4. Conclusions

Microsurgical clipping is a definitive and durable treatment for carotid-ophthalmic tandem aneurysms. The bypass option ought to be considered for very large and giant heavily thrombosed aneurysms.

Anterior clinoidectomy and opening of the distal dural ring are key aspects for paraclinoid TandIAs involving the clinoidal and ophthalmic segment of the ICA. The more distal aneurysm should be clipped first, except for “kissing” aneurysms, for which the risk of intraoperative rupture is higher.

A shorter minimum distance between TandIAs represents an adjunctive technical difficulty for both endovascular therapy and surgery, but counter clipping with “facing” clips and clipping with stacked clips parallel to the ICA allows overcoming this limitation during microsurgical treatment.

## Data Availability

Data are contained within the article.
